# Pharmacodynamic evaluation of commonly prescribed oral antibiotics against respiratory bacterial pathogens

**DOI:** 10.1186/1471-2334-11-286

**Published:** 2011-10-25

**Authors:** Carlos RV Kiffer, Antonio CC Pignatari

**Affiliations:** 1 Laboratório Especial de Microbiologia Clínica, Department of Infectious Diseases, Federal University of São Paulo, São Paulo, Brazil

## Abstract

**Background:**

Upper and lower respiratory tract infections (RTIs) account for a substantial portion of outpatient antibiotic utilization. However, the pharmacodynamic activity of commonly used oral antibiotic regimens has not been studied against clinically relevant pathogens. The objective of this study was to assess the probability of achieving the requisite pharmacodynamic exposure for oral antibacterial regimens commonly prescribed for RTIs in adults against bacterial isolates frequently involved in these processes (*S. pneumoniae*, *H. influenzae*, and *M. catharralis*).

**Methods:**

Using a 5000-subject Monte Carlo simulation, the cumulative fractions of response (CFR), (i.e., probabilities of achieving requisite pharmacodynamic targets) for the most commonly prescribed oral antibiotic regimens, as determined by a structured survey of medical prescription patterns, were assessed against local respiratory bacterial isolates from adults in São Paulo collected during the same time period. Minimal inhibitory concentration (MIC) of 230 isolates of *Streptococcus pneumoniae *(103), *Haemophilus influenzae *(98), and *Moraxella catharralis *(29) from a previous local surveillance were used.

**Results:**

The most commonly prescribed antibiotic regimens were azithromycin 500 mg QD, amoxicillin 500 mg TID, and levofloxacin 500 mg QD, accounting for 58% of the prescriptions. Varied doses of these agents, plus gatifloxacin, amoxicillin-clavulanate, moxifloxacin, and cefaclor made up the remaining regimens. Utilizing aggressive pharmacodynamic exposure targets, the only regimens to achieve greater than 90% CFR against all three pathogens were amoxicillin/amoxicillin-clavulanate 500 mg TID (> 91%), gatifloxacin 400 mg QD (100%), and moxifloxacin 400 mg QD (100%). Considering *S. pneumoniae *isolates alone, azithromycin 1000 mg QD also achieved greater than 90% CFR (91.3%).

**Conclusions:**

The only regimens to achieve high CFR against all three pathogen populations in both scenarios were gatifloxacin 400 mg QD, moxifloxacin 400 mg QD, and amoxicillin-clavulanate 500 mg TID. These data suggest the need for reconsideration of empiric antibiotic regimen selection among adult patients with RTIs in the São Paulo area. Additionally, this type of study could be used to optimize prescribing patterns in specific regions in light of emerging resistance.

## Background

Mortality due to infections represents approximately 85% of all deaths worldwide, and community acquired ones are highly prevalent [1]. Among these, respiratory tract infections (RTIs) are among the most common causes of morbidity in the community worldwide. *Streptococcus pneumoniae *is the most common bacterial cause of upper and lower respiratory tract community infections, particularly pneumonia [2]. Additionally, it is one of the most frequent causative agents in meningitis and bacteremia, as well as the main cause of upper respiratory non invasive infections, such as otitis media and sinusitis [3-5]. Infections caused by *S. pneumoniae *can occur in all age groups, but are more prevalent in children and the elderly [2,6,7], thus recommendations for vaccination in these age groups. Furthermore, other bacterial pathogens are often implicated in respiratory tract infections. *Haemophilus influenzae *is recognized as a frequent cause of acute sinusitis in children and adults as well as pneumonia in developing countries; these respiratory infections are caused most commonly by non-type b strains. Finally, although a less frequent cause of respiratory tract infections, *Moraxella catarrhalis *may be associated with diverse disease conditions, such as laryngitis in adults [8]. It has also been associated with acute exacerbations of chronic obstructive pulmonary disease (COPD), pneumonia in the elderly, and hospital respiratory infections [8]. Since nearly all respiratory infections are treated empirically, knowledge of antibacterial resistance determined periodically with contemporary isolates is valuable.

However, the susceptibility pattern alone might be insufficient to guide optimal antimicrobial therapy, since most of the community acquired infections present a risk of resistance development to a first line antimicrobial drug [1], leading to changes in susceptibility patterns of commonly used agents over time. This resistance pattern modification over time may limit the utility of regulatory clinical studies conducted decades ago. Consequently, prescription patterns may need to evolve, with some compounds requiring dosing adjustments or new formulations in order to maintain efficacy. The increasing burden of antimicrobial resistance coupled with the relative drought on the antimicrobial developmental pipeline has urged for strategies to reduce antibiotic consumption and to maximize treatment approaches [9].

In this setting, pharmacodynamic analysis of established antimicrobial therapies may be a useful approach to predict the successful eradication of specific pathogens causing infection at specific sites [10,11]. Additionally, pharmacodynamic modeling has been an important consideration in the development of guidelines for treatment of infections, including acute otitis media and sinusitis [12-14]. For beta-lactams, the time for which free drug concentrations remains above minimum inhibitory concentration (MIC) as a function of the dosing interval (fT > MIC) is well established to be the pharmacodynamic index predictive of successful response [10]. For fluoroquinolones, the maximum concentration to MIC ratio (Cmax/MIC) or area under the curve to MIC ratio (AUC/MIC) are the pharmacodynamic indices predictive of success [11]. Lastly, for azithromycin, the AUC/MIC appears to be the pharmacodynamic ratio predictive of success [11], due to its long half-life, but clinical studies to demonstrate exposure-response relationships are limited.

The objective of this study was to assess the probability of achieving the requisite pharmacodynamic exposure for oral antibacterial regimens commonly prescribed for respiratory infections in adults against bacterial isolates frequently involved in these processes (*S. pneumoniae*, *H. influenzae*, and *M. catharralis*) in Sao Paulo, Brazil.

## Methods

### Definition of Commonly Prescribed Antimicrobials

A 2006 database (INTE, National Index of Therapeutics and Diseases) from a commercial prescription survey (IMS Health Brasil) was used to determine the antimicrobial prescription pattern of physicians within the city of São Paulo for respiratory tract infections (upper and lower, all inclusive) in adults from January to December 2006. The database is composed of structured prescription data collected through a survey performed with a stratified and representative sample of 399 physicians, divided by city geographical regions and specialty (16 specialties). The survey is conducted every trimester through a structured questionnaire to allow for data capture by each doctor and specific medical visits, patient demographics, week surveyed, type of disease, dose and amount prescribed (volume, formulation and dosage per diagnosis).

For the current study, we determined the mean dose prescribed per day for the most frequent antimicrobials based on total number of mentions (in thousands) for the specific product, number of mentions (in thousands) per dosage, and percent participation of each dosage in formulary for the last 12 months. Antimicrobial drugs prescribed in less than 1% of the population, such as cefuroxime, clarithromycin, and intramuscular ceftriaxone were not included in final analysis.

### Microbiological Data

Microbiological data was extrapolated from a regional 2003-2004 surveillance based on routine bacterial isolates collected from four laboratories located in São Paulo. The isolates were a subset of the referred study, comprising exclusively outpatients older than 16 years of age with respiratory isolates, i.e., sampled from middle ear fluid, sinus fluid, throat swab, sputum, broncho-alveolar lavage (BAL), or nasopharyngeal swab from the referred period. All *S. pneumoniae*, *H. influenzae*, and *M. catharralis *isolates were subjected to MIC determination by microdilution assays according to the respective year's Clinical Laboratory Standard Institute (CLSI) recommendations [15].

### Pharmacokinetics

For the most commonly prescribed oral antibiotics, pharmacokinetic data were obtained from previously published studies in healthy volunteers [16-20]. For studies to be considered, they had to be conducted in at least 10 healthy volunteers, described the assay used to determine drug concentrations and present mean and standard deviation results for the total body clearance in liters per hour (CL), volume of distribution of the central compartment (Vc), absorption rate constant (K01) and other pertinent pharmacokinetic parameters. For amoxicillin, CL and Vc estimates were reported in L/kg/hr and L/kg, respectively, so a patient population with weight of 70 +/- 5 kg was used to scale these parameters during the Monte Carlo simulation. Because a published report with the above characteristics was not identified for cefaclor, we modeled the pharmacokinetics (Winnonlin, Version 5.3) based on a study that provided detailed concentration-time points for each subject [21] and used the model to generate parameters to be applied in the Monte Carlo simulation analyses. As body weight for individual patients was not reported in the cefaclor study, our WinNonLin derived estimates for CL and Vc were in L/hr and L, respectively, and it was not necessary to apply body weight during the Monte Carlo simulation. For the fluoroquinolones and azithromycin, clearance controlled for bioavailability (CL_F) was extrapolated from the pharmacokinetic studies.

Simulated distributions for all pharmacokinetic parameters were consistent with log-Gaussian distributions in accordance with the data inputted into the models (Table 1).

**Table 1:** Summary of pharmacokinetic parameters for antimicrobials used in the Monte Carlo simulation

	Pharmacokinetic Parameter (mean ± SD)					
	
Antibiotic	CLT (L/hr)	Vc (L)	Fraction unbound (%)*	**Intercompartment Rate Constants (h**^**-1**^**)**		**Absorption Rate Constant (h**^**-1**^**)**
				K12	K21	K01
Amoxicillin ± clavulanate	22.2677 ± 5.8091	14.0622 ± 5.5932	80 ± 2	1.77 ± 2.27	1.91 ± 1.12	0.93 ± 0.49
Cefaclor	43.1513 ± 13.0045	29.3195 ± 8.3441	75 ± 5	0.45 ± 0.12	1.15 ± 0.35	2.61 ± 0.78
	**CL_F (L/hr)**					

Azithromycin	122 ± 33	---	---			
Levofloxacin	10.5 ± 1.48	---	62-76			
Gatifloxacin	12.54 ± 1.27	---	78-82			
Moxifloxacin	14.9 ± 1.18	---	49.5-70			

* Fraction unbound estimates simulated as a mean (SD) Gaussian distribution for amoxicillin, co-amoxiclav, and cefaclor. Estimates simulated as a range for the FQ and azithromycin, where each value in the range has an equal likelihood of occurring.

CLT = total body clearance reported from 2-compartment model; Vc = volume of the central compartment reported from 2-compartment model; K12 = intercompartment rate constant between first and second compartments; K21 = intercompartment rate constant between second and first compartments; K01 = absorption rate constant; CL_F = clearance accounting for bioavailability reported in pharmacokinetic analyses.

### Pharmacodynamic Analyses

A 5000-patient Monte Carlo simulation (Crystal Ball, 2000) was conducted to calculate estimates of *f*T > MIC or the AUC/MIC ratio for each antibiotic regimen/bacterial population combination. The dosage regimens chosen were based on the most common regimens prescribed according to the survey database. Pharmacodynamic exposures, as measured by *f*T > MIC, were simulated for oral regimens of amoxicillin 500 mg three times daily (TID) and 875 mg twice daily (BID), amoxicillin-clavulanate 500/125 mg q8h and 875/125 mg BID, and cefaclor 500 mg, 750 mg, 250 mg, and 375 mg BID against all pathogens (except for cefaclor, only simulated against *S. pneumoniae*). For amoxicillin, amoxicillin-clavulanate, and cefaclor, a two-compartment pharmacokinetic model with lag time was used to simulate steady-state concentration time profiles. Pharmacodynamic exposures, as measured by free drug AUC/MIC ratio, were simulated for levofloxacin 500 mg and 250 mg once daily (QD), gatifloxacin 400 mg QD, moxifloxacin 400 mg QD (all pathogens). Pharmacodynamic exposures, as measured by the total drug 24-hours AUC/MIC ratio, were modeled for azithromycin 500 mg and 1000 mg QD (all pathogens). For the fluoroquinolones and for azithromycin, AUC was calculated as the daily dose divided by CL_F.

Values for *f*T > MIC and AUC/MIC were plotted on frequency curves for further analysis. The probabilities of obtaining a *f*T > MIC of 20%, 30%, 40%, 50%, 60%, 70%, 80%, 90%, and 100% were calculated for all the beta-lactams at increasing MICs in doubling dilutions. For comparative purposes, two scenarios were built for beta-lactams and for azithromycin: 1) aggressive pharmacodynamic breakpoints were defined as requiring 50% *f*T > MIC for all beta-lactams and total AUC/MIC ≥ 25 [22] for azithromycin; 2) conservative pharmacodynamic breakpoints were defined as requiring 30% *f*T > MIC for all beta-lactams and total AUC/MIC ≥ 10 [23] for azithromycin. In both scenarios, the probability of achieving a *f*AUC/MIC ratio of at least 33.7 was calculated for fluoroquinolones [24]. These probabilities of target attainment were applied to the MIC distributions for the above pathogens to calculate the cumulative fraction of response (CFR).

## Results

### Most commonly prescribed antimicrobial agents

Table 2 provides the antimicrobials and dosing regimens most frequently prescribed for community RTIs (excluding pediatric formulations) and the relative frequency of prescription for each presentation.

**Table 2:** Most frequently prescribed antimicrobial regimens for community RTIs with relative prescription frequency.

ANTIMICROBIAL	Prescribed Dosing Regimen	% Relative prescription frequency per drug formulation
AZITHROMICIN	500 mg QD	27.4%
AMOXICILLIN	500 mg TID	15.4%
LEVOFLOXACIN	500 mg QD	14.7%
AMOXICILLIN	875 mg BID	6.5%
GATIFLOXACIN	400 mg QD	6.3%
AZITHROMICIN	1000 mg QD	6.0%
AMOXICILLIN-CLAVULANIC ACID	875 mg BID	5.8%
LEVOFLOXACIN	250 mg QD	5.1%
AMOXICILLIN-CLAVULANIC ACID	500 mg TID	4.7%
MOXIFLOXACIN	400 mg QD	3.5%
CEFACLOR	500 mg BID	0.8%
CEFACLOR	750 mg BID	0.8%
CEFACLOR	250 mg BID	0.5%
CEFACLOR	375 mg BID	0.4%

### Microbiology

Table 3 shows MIC distributions for 230 total isolates of *S. pneumoniae *(103), *H. influenzae *(98), and *M. catharralis *(29) from a local prevalence study. According to present CLSI criteria [15], *S. pneumoniae *showed 100% susceptibility to penicillin, 91.3%to azithromycin, and 100%to the fluoroquinolones (levo, moxi, and gatifloxacin). As for *H. influenzae*, susceptibility to penicillin was 87.7% and 100% to azithromycin. *M. catharralis *showed 100% of MICs to amoxicillin above 1 mcg/mL.

**Table 3:** MIC distributions for *S. pneumoniae*, *H. influenza*, and *M. catharralis *included in the study

	Percentage (%) of bacteria at each MIC value (mg/L)													
	
Bacterial group (n) and antimicrobial	0.008	0.016	0.032	0.06	0.125	0.25	0.5	1	2	4	8	16	32	> 32
*S. pneumoniae *(103)														
Penicillin	2.9	27.2	24.3	18.4	7.8	5.8	1.0	9.7	2.9	0.0	0.0	0.0	0.0	0.0
Amoxicillin	0.0	1.0	64.1	17.5	2.9	1.9	1.0	1.0	7.8	1.9	1.0	0.0	0.0	0.0
Co-amoxiclav	0.0	1.0	64.1	17.5	2.9	1.9	1.0	1.0	7.8	1.9	1.0	0.0	0.0	0.0
Cefaclor	0.0	0.9	0.0	1.9	4.6	36.1	37.0	5.6	1.9	1.9	0.9	0.9	5.6	2.8
Azithromycin	0.0	0.0	7.8	63.1	20.4	0.0	0.0	0.0	0.0	0.0	1.9	1.0	0.0	5.8
Levofloxacin	0.0	0.0	0.0	0.0	0.0	0.0	14.6	80.6	4.9	0.0	0.0	0.0	0.0	0.0
Gatifloxacin	0.0	0.0	0.0	0.0	2.9	79.6	17.5	0.0	0.0	0.0	0.0	0.0	0.0	0.0
Moxifloxacin	0.0	0.0	0.0	10.7	71.8	17.5	0.0	0.0	0.0	0.0	0.0	0.0	0.0	0.0

*H. influenzae *(98)														
Penicillin	0.0	0.0	0.0	0.0	55.1	16.3	11.2	5.1	0.0	0.0	3.1	4.1	5.1	0.0
Amoxicillin	0.0	0.0	0.0	0.0	7.1	51.0	19.4	8.2	2.0	3.1	0.0	0.0	9.2	0.0
Co-amoxiclav	0.0	0.0	0.0	0.0	9.2	44.9	30.6	12.2	3.1	0.0	0.0	0.0	0.0	0.0
Azithromycin	0.0	0.0	0.0	0.0	3.1	5.1	24.5	44.9	21.4	1.0	0.0	0.0	0.0	0.0
Levofloxacin	30.6	66.3	2.0	1.0	0.0	0.0	0.0	0.0	0.0	0.0	0.0	0.0	0.0	0.0
Gatifloxacin	0.0	96.9	2.0	1.0	0.0	0.0	0.0	0.0	0.0	0.0	0.0	0.0	0.0	0.0
Moxifloxacin	11.2	67.3	18.4	2.0	1.0	0.0	0.0	0.0	0.0	0.0	0.0	0.0	0.0	0.0

*M. catharralis *(29)														
Amoxicillin	0.0	0.0	0.0	0.0	0.0	0.0	0.0	6.9	6.9	13.8	24.1	27.6	20.7	0.0
Co-amoxiclav	0.0	0.0	0.0	0.0	51.7	41.4	6.9	0.0	0.0	0.0	0.0	0.0	0.0	0.0
Azithromycin	0.0	0.0	0.0	96.6	0.0	3.4	0.0	0.0	0.0	0.0	0.0	0.0	0.0	0.0
Levofloxacin	0.0	0.0	93.1	6.9	0.0	0.0	0.0	0.0	0.0	0.0	0.0	0.0	0.0	0.0
Gatifloxacin	0.0	20.7	75.9	3.4	0.0	0.0	0.0	0.0	0.0	0.0	0.0	0.0	0.0	0.0
Moxifloxacin	0.0	0.0	34.5	65.5	0.0	0.0	0.0	0.0	0.0	0.0	0.0	0.0	0.0	0.0

MIC = minimum inhibitory concentration; Co-amoxiclav = amoxillin-clavulanate

### Pharmacokinetic parameters

Table 1 shows a summary of pharmacokinetic parameters for antimicrobials used in the Monte Carlo simulations.

### Pharmacodynamic target attainment

Table 4 lists CFR results using the aggressive pharmacodynamic exposure targets (scenario 1) for each prescribed antimicrobial regimen against each of the outpatient respiratory bacteria. Table 5 lists CFR results using the conservative pharmacodynamic exposure targets (scenario 2) for each prescribed antimicrobial regimen against each of the outpatient respiratory bacteria.

**Table 4:** Scenario 1: Cumulative fraction of response (CFR) for achieving aggressive* pharmacodynamic indice exposures for prescribed antimicrobial regimens against outpatient respiratory isolates

ANTIBIOTIC	Dosing Regimen	*S. pneumoniae*	*H. influenzae*	*M. catharralis*
AMOXICILLIN	875 mg BID	88.2%	69.1%	4.8%
AMOXICILLIN	500 mg TID	91.2%	82.1%	7.4%
AMOXICILLIN-CLAVULANIC ACID	875 mg BID	88.2%	76.1%	88.8%
AMOXICILLIN-CLAVULANIC ACID	500 mg TID	91.2%	91.9%	98.5%
AZITHROMICIN	1000 mg QD	91.3%	46.8%	99.6%
AZITHROMICIN	500 mg QD	76.50%	7.8%	81.4%
CEFACLOR	750 mg BID	17.0%	--	--
CEFACLOR	500 mg BID	9.8%	--	--
CEFACLOR	375 mg BID	6.1%	--	--
CEFACLOR	250 mg BID	2.6%	--	--
GATIFLOXACIN	400 mg QD	100.0%	100.0%	100.0%
LEVOFLOXACIN	500 mg QD	51.4%	100.0%	100.0%
LEVOFLOXACIN	250 mg QD	6.6%	100.0%	100.0%
MOXIFLOXACIN	400 mg QD	100.0%	100.0%	100.0%

* Beta-lactams = 50% *f*T > MIC; Azithro = total AUC/MIC ≥ 25; Fluoroquinolones *f*AUC/MIC ≥ 33.7

**Table 5:** Scenario 2: Cumulative fraction of response (CFR) for achieving conservative* pharmacodynamic indice exposures for prescribed antimicrobial regimens against outpatient respiratory isolates

ANTIBIOTIC	Dosing Regimen	*S. pneumoniae*	*H. influenzae*	*M. catharralis*
AMOXICILLIN	875 mg BID	95.5%	86.9%	14.6%
AMOXICILLIN	500 mg TID	96.3%	87.8%	14.8%
AMOXICILLIN-CLAVULANIC ACID	875 mg BID	95.5%	97.8%	99.7%
AMOXICILLIN-CLAVULANIC ACID	500 mg TID	96.3%	99.4%	100.0%
AZITHROMICIN	1000 mg QD	91.8%	99.2%	100.0%
AZITHROMICIN	500 mg QD	91.3%	67.9%	99.9%
CEFACLOR	750 mg BID	58.6%	--	--
CEFACLOR	500 mg BID	45.5%	--	--
CEFACLOR	375 mg BID	35.4%	--	--
CEFACLOR	250 mg BID	22.0%	--	--
GATIFLOXACIN	400 mg QD	100.0%	100.0%	100.0%
LEVOFLOXACIN	500 mg QD	51.4%	100.0%	100.0%
LEVOFLOXACIN	250 mg QD	6.6%	100.0%	100.0%
MOXIFLOXACIN	400 mg QD	100.0%	100.0%	100.0%

* Beta-lactams = 30% *f*T > MIC; Azithro = total AUC/MIC ≥ 10; Fluoroquinolones *f*AUC/MIC ≥ 33.7

## Discussion

The present study evaluated the probability of achieving pharmacodynamic exposures for the most frequently prescribed oral antimicrobial regimens used to treat respiratory infections in adults in the São Paulo region against the most commonly isolated bacterial pathogens. This is the first multidrug comparison of oral antibiotics against clinically relevant RTI pathogens in adults. The antimicrobial regimens simulated were extracted from a commercial database survey applied to a representative medical population of the São Paulo region in 2006. Additionally, the infectious pathogens (*S. pneumoniae*, *H. influenzae*, and *M. catharralis*) were isolated from the same geographical area (city of São Paulo) within a similar, albeit slightly earlier, time period (2003-2004). The pathogens were part of a previous local surveillance study collected from four (4) centers located in São Paulo during a two (2) year study. Only isolates from adult (> 16 years-old) outpatients with respiratory infections (sampled from middle ear fluid, sinus fluid, throat swab, sputum, broncho-alveolar lavage (BAL), or nasopharyngeal swab) were included in the present analysis. Since nearly all RTIs are treated empirically, knowledge of antibacterial resistance determined periodically is valuable. In this study, the bacterial isolates included were in general representative for RTIs in the adult population during years 2003 and 2004. The total 230 isolates were apparently significant for this population [25,26], with less *M. catharralis *isolates.

We detected that different formulations (i.e. doses) of azithromycin, amoxicillin, and levofloxacin accounted for 85.7% of the top prescribed respiratory drugs. Azithromycin 500 mg QD ranked first regimen (27.4%) for RTIs in the 2006 period. We did not detect high dose amoxicillin and/or co-amoxiclav (1000 mg TID) in the prescription survey. It is important to notice that both high dose beta-lactams are present in recommendations issued by the Infectious Disease Society of America (IDSA)/American Thoracic Society (ATS) for community acquired pneumonia [27], but the survey was conducted prior to the release of these recommendations. It should also be noted that, although the prescribing pattern in São Paulo only has local representation, prescription surveys may be useful to guide future pharmacodynamic modeling in diverse environments.

As for the pharmacodynamic targets adopted, we do acknowledge the existence of a large discrepancy referring to what should be considered an aggressive management strategy, particularly for beta-lactams. Although bactericidal targets for beta-lactams have been widely disputed, target attainment at various T > MIC exposures were modeled. However, the respective figure for various T > MIC exposure targets was not included in the present paper for better clarity. Also, it is noteworthy to mention that prescription data was from 2006 and microbiological data from was from 2003-2004. Although there may have been changes in both resistance and prescription patterns over time, periods were close enough in order to achieve the main purpose of the study, which was to shed light on commonly prescribed oral agents and their potential activity or lack thereof. Additionally, the study could serve as a model to be used within areas with different prescribing habits.

Based on the premises that antimicrobial survey data was indicative of prescription and usage, and that microbiological data was representative of RTIs in adults, both in similar period and area, simulation was performed using previously published pharmacodynamic targets. Two hypothetic scenarios were used: scenario 1, with more aggressive pharmacodynamic exposure targets for beta-lactams (50% *f*T > MIC) and for azithromycin (total AUC/MIC ≥ 25); and scenario 2, with more conservative targets (Beta-lactams = 30% *fT *> MIC; azithromycin = total AUC/MIC ≥ 10). Although appropriate pharmacodynamic targets for azithromycin are not well established, the different scenarios presented aimed at partially addressing this issue.

In scenario 1, as shown in table 4, only gatifloxacin 400 mg QD and moxifloxacin 400 mg QD achieved 100% CFR against all three pathogens, while amoxicillin-clavulanate (500/125 mg TID) and azithromycin 1000 mg QD both achieved above 90%. All other antimicrobial regimens achieved below 90% CFR against at least one pathogen in scenario 1. Scenario 2, shown in table 5, with a more conservative pharmacodynamic profile, demonstrated that CFR for gatifloxacin 400 mg QD and moxifloxacin 400 mg QD were again 100% against all pathogens. However, this time, amoxicillin-clavulanate in both dosing formulations (500/125 mg TID and 875/125 mg BID) achieved above 90% CFR for all pathogens. All other antimicrobial regimens achieved below 90% CFR for at least one pathogen in scenario 2. It is relevant, though, that azithromycin 1000 mg QD achieved high CFR against all pathogens in this scenario, but azithormycin 500 mg QD had a worst performance against *H. influenzae *(67.9%).

Also of importance, levofloxacin 500 mg QD did not perform as well as gatifloxacin and moxifloxacin against *S. pneumoniae*, with a CFR of 51.4%. It has been previously demonstrated that gatifloxacin or moxifloxacin have a higher probability of achieving the AUC/MIC target than does levofloxacin [28]. Although the present study did not evaluate the levofloxacin 750 mg QD regimen, it has also been established that levofloxacin administered at a dose of 750 mg QD has a higher CFR and improved bacteriological outcome against *S. pneumoniae *than the 500 mg QD regimen [29,30]. It seems that for the environment studied, the levofloxacin 500 mg QD regimen is not adequate against *S. pneumoniae*. However, it is important to note that most studies [28-31] demonstrated a poor probability of target attainment for the levofloxacin 500 mg QD regimen when MICs are 1 mcg/ml or more. Most of the isolates evaluated (80.6%) had MICs of 1 mcg/mL. However, another possible explanation for the low CFR found for levofloxacin lies on the clearance utilized to simulate its exposure. The present study used healthy volunteer data and it has been previously shown that levofloxacin clearance is reduced in patients compared with healthy volunteers, leading to higher AUCs in the patient population [28,29].

Pichichero et al [32] developed a similar strategy, while assessing the probability of achieving requisite pharmacodynamic exposures through Monte Carlo simulation for commonly used antimicrobials to treat children with bacterial RTIs against contemporary *H. influenzae*. A relevant conclusion in their article was that cefpodoxime, ceftibuten, and amoxicillin/clavulanic acid were the most likely antimicrobials to achieve optimal in vivo exposures in children with *H. influenzae *infections. In the present study we aimed at the adult population and their commonly prescribed antimicrobials. Also, the regimens were simulated against the most frequent bacterial pathogens causing RTIs, i.e. *S. pneumoniae*, *H. influenzae*, and *M. catharralis*. The use of the IMS database seems significant from the clinical viewpoint, since it is an alternative to obtain an estimation of population antimicrobial usage patterns. Nevertheless, it should not be understood as a definite result, since prescription patterns vary from place to place and do not necessarily represent usage. However, it could apparently be used as a possible indication of significant prescription data within specific environments.

## Conclusion

Overall, the fluoroquinolones, gatifloxacin and moxifloxacin, attained the highest CFR (100%) against all pathogens studied. Across all pathogens, co-amoxiclav 500 mg TID and 875 mg BID and azithromycin 1000 mg QD (in the conservative scenario) were above 90% also. Both cefaclor and low dose levofloxacin (250 mg QD) should be avoided to treat RTIs in this environment, due to their low performance against major RTI pathogens (and against *S. pneumoniae *for cefaclor). The present data suggest the need for reconsideration of empiric antibiotic regimen selection among adult patients with respiratory tract infections in the São Paulo area. It is our understanding that this type of study can be used to optimize prescribing patterns in specific regions in light of emerging resistance.

## Competing interests

The authors declare no competing interests related to this publication. This Research was supported by FAPESP (State Agency for Research Funding) Research Grant 2009/13825-2 (http://www.fapesp.br/en/). FAPESP had no interference in the writing of the manuscript or the decision to submit it for publication. No authors have been paid to write this article by a pharmaceutical company or other agency. I, as corresponding author, had full access to all the data in the study and had final responsibility for the decision to submit for publication.

## Authors' contributions

CRVK designed, collected, executed and performed the analysis, reviewed the data, and wrote the report. ACCP supervised the analysis, critically reviewed the experiment and helped interpreting and discussing the data. All authors read and approved the final manuscript.

## Pre-publication history

The pre-publication history for this paper can be accessed here:

http://www.biomedcentral.com/1471-2334/11/286/prepub
